# Physiological Impact of Abnormal Lipoxin A_4_ Production on Cystic Fibrosis Airway Epithelium and Therapeutic Potential

**DOI:** 10.1155/2015/781087

**Published:** 2015-03-19

**Authors:** Gerard Higgins, Fiona Ringholz, Paul Buchanan, Paul McNally, Valérie Urbach

**Affiliations:** ^1^National Children's Research Centre, Crumlin, Dublin 12, Ireland; ^2^Royal College of Surgeons in Ireland, Beaumont Hospital, Dublin 9, Ireland; ^3^Institut National de la Santé et de la Recherche Médicale, U845, Faculté de Médecine Paris Descartes, Site Necker, 156 rue Vaugirard, 75015 Paris, France

## Abstract

Lipoxin A_4_ has been described as a major signal for the resolution of inflammation and is abnormally produced in the lungs of patients with cystic fibrosis (CF). In CF, the loss of chloride transport caused by the mutation in the cystic fibrosis transmembrane conductance regulator (CFTR) Cl^−^ channel gene results in dehydration, mucus plugging, and reduction of the airway surface liquid layer (ASL) height which favour chronic lung infection and neutrophil based inflammation leading to progressive lung destruction and early death of people with CF. This review highlights the unique ability of LXA_4_ to restore airway surface hydration, to stimulate airway epithelial repair, and to antagonise the proinflammatory program of the CF airway, circumventing some of the most difficult aspects of CF pathophysiology. The report points out novel aspects of the cellular mechanism involved in the physiological response to LXA_4_, including release of ATP from airway epithelial cell via pannexin channel and subsequent activation of and P2Y11 purinoreceptor. Therefore, inadequate endogenous LXA_4_ biosynthesis reported in CF exacerbates the ion transport abnormality and defective mucociliary clearance, in addition to impairing the resolution of inflammation, thus amplifying the vicious circle of airway dehydration, chronic infection, and inflammation.

## 1. Lipoxin A_4_

### 1.1. Lipoxin A_4_ and Eicosanoid Class Switching

Lipoxin A_4_ (LXA_4_) belongs to a class of newly identified specialised proresolution lipid mediators playing a central role in the resolution of inflammation which results from the sequential production of characteristic eicosanoids in a process termed “class switching” [1, 2]. Prostaglandins are biosynthesized early, initiating the acute inflammatory response. Then Leukotrienes typified by Leukotriene B_4_ (LTB_4_) play a role in the amplification and propagation of inflammation [1] acting in concert with the peptide Interleukin 8 (IL8) as a potent neutrophil chemoattractant [3, 4]. Both LTB_4_ and IL8 are negatively correlated with pulmonary function in CF. LXA_4_ is the first eicosanoid expressed in the active resolution phase of inflammation [5] followed by biosynthesis of the Resolvins and Protectins. LTB_4_ and LXA_4_ are closely related metabolites of arachidonic acid and can be synthesised from a common unstable intermediate [3].

### 1.2. Lipoxin A_4_ Synthesis

LXA_4_ is produced by multistep enzymatic process resulting from lipoxygenase (LO) activities in different cell types [6]. Neutrophils [7], eosinophils [8], alveolar macrophages [9], platelets [10], or airway epithelial cells [11] express different LO which act in sequence in LXA_4_ biosynthesis [3, 12].

**Figure 1:**
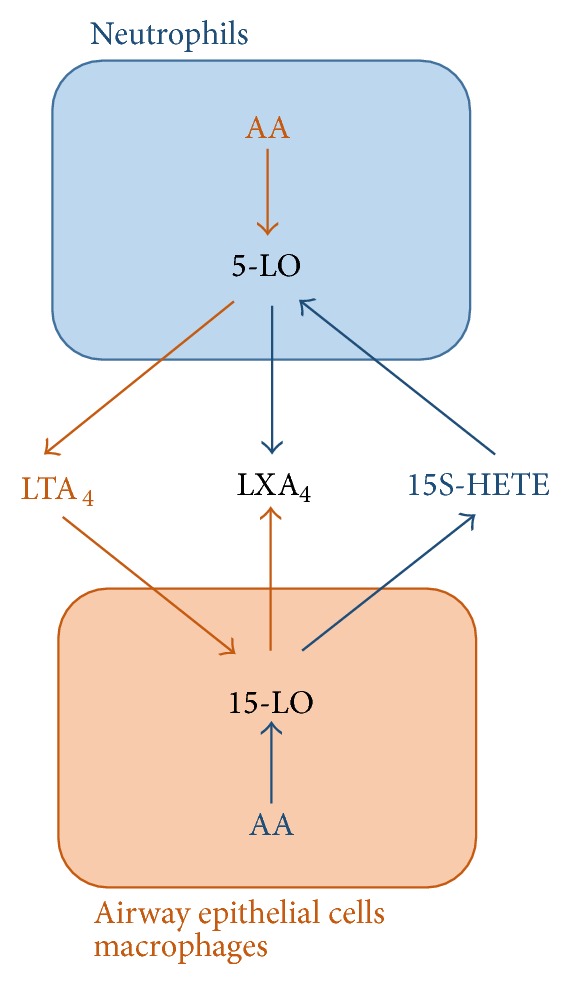
Lipoxin A_4_ biosynthesis by trans-cellular cooperation in the airways. The neutrophil donates LTA_4_ intermediate formed by the action of 5 lipoxygenase (5-LO) on arachidonic acid (AA) to the acceptor airway epithelial cell or alveolar macrophage whereby 15 lipoxygenase (15-LO) catalyses LXA_4_ formation (brown arrows). Airway epithelial cell or alveolar macrophage 15-LO activity catalyses the conversion of AA to 15S-HETE which is donated to the acceptor neutrophil and converted to LXA_4_ by 5-LO catalysis (blue arrows).

Two main pathways will result in LXA_4_ synthesis. One involves lipoxygenation of arachidonic acid by 15-LO in macrophages and epithelial cells. The 5-LO expressed by neutrophils can then utilise the 15(S)-hydroxyeicosatetranoic acid (15S-HETE) released as a substrate to synthesize LXA_4_ [7] (Figure 1, blue arrows). Alternatively, platelet 12-LO [10] and macrophage or epithelial 15-LO [13, 14] are each able to transform Leukotriene A_4_, released by neutrophils, into LXA_4_ (Figure 1, brown arrows). The activity of 15-LO promotes LXA_4_ biosynthesis and blocks leukotriene biosynthesis, both as a result of 15-LO products competing for flux at the 5-LO level and by diversion of the intermediate Leukotriene A_4_ away from LTB_4_ towards LXA_4_ biosynthesis [1, 11, 15].

### 1.3. Lipoxin A_4_ Anti-Inflammatory Actions

The anti-inflammatory action of LXA_4_ is mainly mediated by the formyl-peptide receptor 2 (FPR2) which is one member of a subgroup of receptors linked to inhibitory G-proteins, also called ALX [16, 17]. FPR2 receptor activation by specific agonists results in transient Ca^2+^ flux, phosphorylation of extracellular signal regulated kinases (ERK), and chemotaxis [18]. The molecular and pharmacological characterization of FPR2 receptor have been previously reviewed [19, 20]. Briefly, the seventh transmembrane domain of the FPR2 receptor is essential for LXA_4_ recognition, whereas the additional regions of the receptor (e.g., extracellular loops) are required for high affinity binding of the peptide ligands [17, 19, 20]. LXA_4_ also interacts directly with the cysLTI receptor to transduce signals that prevent the proinflammatory response and contributes to the active resolution of inflammation [18, 21].

LXA_4_ inhibits neutrophil effector functions [5] and in particular inhibits LTB_4_ induced neutrophil transmigration [22–24]. LXA_4_ suppresses IL8 production by leukocytes and bronchial epithelial cells including airway epithelial cells from patients with cystic fibrosis [25–28]. Mice treated with analogues of LXA_4_ and subsequently challenged with* P. aeruginosa* contained the bacterial challenge more effectively [29]. LXA_4_ affects leukocytes in a cell type specific manner, inhibiting the activation of polymorphonuclears (PMNs) and eosinophils whilst activating monocytes and macrophages. PMN recruitment is a multistep process that involves chemotaxis, adhesion, and transmigration. In* in vitro* models LXA_4_, LXB_4_, and ATLS inhibit PMN chemotaxis in response to the chemoattractant LTB_4_ and inhibit eosinophil responses to platelet activating factor. Stimulation of macrophages with LXA_4_ significantly enhances phagocytosis of apoptotic PMN, suggesting that LXA_4_ can promote the clearance of apoptotic leukocytes by macrophages at an inflammatory site [30, 31].

## 2. Cystic Fibrosis

### 2.1. Cystic Fibrosis Disease and the CF Gene

Cystic fibrosis (CF) is the most common lethal genetic disorder in Caucasians caused by a mutation in the gene encoding the cystic fibrosis transmembrane conductance regulator (CFTR). The disease was first characterised in 1938 by Andersen who described the cystic fibrosis of the pancreas and correlated it with the lung and intestinal disease that occurs in CF [32]. In 1953, the observation of excessive salt loss in the sweat of CF patients was noted; however, it was not until 1983 that it was first shown that sufferers of CF displayed abnormal chloride transport. This discovery was not sufficient for the identification of the defective protein in CF patients. In 1985 polymorphic markers associated with the disease were identified and finally the CFTR gene was identified [33–35].

The CFTR protein is principally expressed in the apical membranes of epithelia where it acts as an anion channel providing a pathway for Cl^−^ and bicarbonate (HCO_3_^−^) movement, controlling the rate of fluid flow, and also regulating the function of other ion channels and transporters in epithelial cells [36, 37]. A number of different CFTR mutations have been identified that lead to differing outcomes in terms of protein synthesis, trafficking, regulation, and CFTR levels within the cell [38, 39]. CFTR is abundantly expressed in epithelial cell membranes and, as such, CF disease particularly affects epithelial sites: the submucosal glands, airway surface epithelium [40], pancreatic ductal epithelium, the epithelium of the crypts of Lieberkuhn throughout the gastrointestinal tract [41], the epithelium of sweat glands [42], the epithelium of the developing genital ducts, adult epididymis and vas deferens, and the cervical and the uterine epithelial surfaces [43, 44]. However, there are exceptions among epithelial tissues where CF related dysfunction is not prominent, such as kidney collecting ducts, the epithelium of Burners gland, and the submucosal glands of the duodenum [44]. The major clinical features of CF are chronic pulmonary disease, exocrine pancreatic insufficiency, and male infertility. CF lung disease reflects the failure of airway defence against chronic bacterial infection, leading to an aggravated immune response, bronchial epithelial remodelling, and ultimately lung destruction. The progressive lung destruction is the main cause of morbidity and mortality in CF [45, 46]. Whilst it was initially believed that the pulmonary complaints associated with CF were directly related to the CFTR dysfunction in epithelial cells, it is now recognised that other cell types including neutrophils [47, 48], macrophages [49, 50], and dendritic cells [51] are directly affected by the absence or dysfunctional CFTR.

### 2.2. Abnormal Production of Lipoxin A_4_ in Cystic Fibrosis

In addition to CFTR dysfunction, other abnormalities have been described in chronically inflamed and infected CF airways, including intrinsic proinflammatory properties, amplified inflammatory responses to infections, and reduced bacterial clearance. More specifically, the levels of LXA_4_ have been reported to be decreased in CF, like in other chronic airway inflammatory diseases such as asthma [29, 52–55]. A significant suppression in LXA_4_/neutrophil ratios in bronchoalveolar lavages (BAL) fluid of patients with CF compared with pulmonary inflammatory controls was reported [29, 56]. Furthermore, in paediatric CF BAL even in the absence of infection, the ratio of LXA_4_ to LTB_4_ is depressed and this correlates with a significant lower level of 15 LO-2 transcripts in CF BAL [2]. A decreased proportion of proresolving compounds (LXA_4_) compared to proinflammatory (LTB_4_) is associated with decreased lung function parameters [57]. In addition,* in vitro* studies support a role for CFTR in LXA_4_ production. The inhibition of CFTR reduces LXA_4_ synthesis by 50% during platelets/PMN coincubation by inhibiting the lipoxin synthase activity of platelets 12-LO. This correlated with the observation that platelets from patients with CF generated 40% less LXA_4_ compared to healthy subjects [58]. The decreased LXA_4_ production in CF provides a mechanistic explanation of the failure to actively resolve acute airway inflammation seen in these patients.

## 3. Regulation of Ion Transport and Airway Surface Liquid Layer in Cystic Fibrosis

### 3.1. Abnormal Ion and Fluid Transport in Cystic Fibrosis

**Figure 2:**
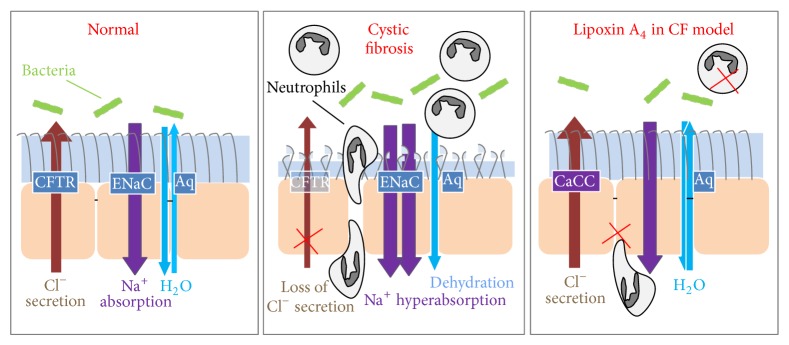
In normal airways the airway surface liquid layer (ASL) provides an adequate mucociliary clearance which is maintained by a combination of Cl^−^ secretion through the cystic fibrosis transmembrane conductance regulator (CFTR), Na^+^ absorption via the epithelial sodium channel (ENaC), and water transport through a paracellular pathway and membrane bound aquaporins (Aq). In CF, a defective CFTR leads to loss of Cl^−^ secretion and Na^+^ hyperabsorption. The concomitant dehydration of the airway lumen favours bacterial infection and inflammation (mainly neutrophilic). LXA_4_ mediates an increase in ASL height and restores it to normal levels in CF bronchial epithelium. LXA_4_ also increase tight junction formation, reestablishing the epithelial barrier function. Taken together this work provides evidence for LXA_4_ as potentially a new therapy for CF patients.

The lung must continually defend itself against bacteria that deposit on the airway surfaces during normal tidal breathing. Mucus clearance is a primary form of pulmonary defence and the efficiency of mucociliary clearance in large part depends upon the volume of the airway surface liquid layer (ASL). The ASL allows for mucus containing foreign bodies to be transported away from the lung to the oropharynx where it is either expelled from the body or swallowed and destroyed by the gut. The ASL provides a low viscosity solution allowing free ciliary beat and mucus transport [59]. The normal hydration of the airway surface is maintained (in the highly water permeable airway epithelium) by active ion-transport controlling the quantity of salt (NaCl) delivered to airway surfaces, with water following passively by osmosis [60]. The NaCl concentration of the airway surface liquid is tightly regulated in normal airway epithelia by the epithelial sodium channel (ENaC) mediated Na^+^ absorption and Cl^−^ secretion. Cl^−^ is secreted by epithelial cells via the apical CFTR Cl^−^ channel and calcium activated Cl^−^ channels, with Cl^−^ entering the cell through the Na^+^–K^+^–2Cl^−^ cotransporter located in the basolateral membrane. Regulation of Cl^−^ secretion determines the net transport of ions across the epithelium and hence the mass of salt on the epithelial surfaces. CFTR was also found to regulate ENaC suggesting that CFTR acted both as a Cl^−^ channel and as a regulator of other ion transport processes. In CF, mutations of the CFTR gene result in defective Cl^−^ secretion and Na^+^ hyperabsorption by airway epithelia [61, 62]. Studies in CF airway epithelium cultures, transgenic mice, and people with CF suggest that the initiating event in CF airway disease is a reduced ASL volume resulting from dehydration. This dehydration leads to reduced mucus clearance, adhesion of mucus to airway surfaces, and chronic bacterial infection of the lung (Figure 2). The chronic bacterial infection leads to an aggravated immune response, bronchial epithelial remodelling, and ultimately lung destruction [59, 63–70].

### 3.2. Lipoxin A_4_ Restores Fluid Transport in Cystic Fibrosis

One of the greatest challenges of fundamental research into reversing the CF defect in the lung has been to design a strategy to overcome the absence of functional CFTR by stimulating chloride secretion via alternative pathways, thus restoring airway hydration and mucociliary clearance. This can be achieved via the stimulation of calcium activated Cl^−^ by agents that raise the intracellular concentration of calcium. Yet, this strategy has been plagued by the side effects of the amplification of the calcium-dependent proinflammatory response resulting in undesirable activation of NF*κ*B. In addition to its anti-inflammatory properties, LXA_4_ stimulates a rapid and transient intracellular Ca^2+^ increase in normal and CF bronchial epithelial cells expressing the FPR2 receptor [71, 72]. This intracellular calcium signal is mainly due to calcium mobilisation from intracellular calcium stores in non-CF airway epithelial cells and due to calcium entry and intracellular calcium release in CF airway epithelial cells. In both, non-CF and CF bronchial epithelia, LXA_4_ stimulates whole-cell Cl^−^ currents which are inhibited by NPPB (calcium-activated Cl^−^ channel inhibitor) and BAPTA-AM (chelator of intracellular Ca^2+^) but not by CFTRinh-172 (CFTR inhibitor) [71, 72]. Furthermore, in models of fully differentiated bronchial epithelia derived from primary culture of bronchial brushings from patients with CF and cultured under air-liquid interface, LXA_4_'s effects on ion transport result in an increase of the airway surface liquid (ASL) layer height. LXA_4_ exerts this effect on the ASL dynamics via the FPR2 receptor. The sustained increase in ASL height induced by LXA_4_ in non-CF and CF bronchial epithelia results from inhibition of amiloride-sensitive Na^+^ absorption and stimulation of an intracellular calcium signal and Ca^2+^-activated Cl^−^ secretion independent from CFTR [72, 73]. LXA_4_ thus restores Cl^−^ secretion and normal ASL height both central to the pathophysiology of CF airway disease, highlighting a role for LXA_4_ in the restoration of normal innate immune defence (Figure 2).

## 4. Nucleotides and CF Airway Disease

### 4.1. Regulation of ASL and Mucociliary Clearance by Nucleotides

Mason et al. first proposed that extracellular ATP regulates ion transport rates when added to either the apical or basolateral surface of human airway epithelium and found that these effects appear to be mediated by cell surface receptors that respond to ATP by regulating ion transport rates through the release of Ca^2+^ from internal stores and extracellular Ca^2+^ influx [74]. As agonists were being screened to restore Cl^−^ and fluid secretion in CF airway epithelium, nucleotide agonists emerged quickly as stimulants of Cl^−^ and fluid secretion independent of CFTR. Knowles et al. showed that extracellular nucleotides stimulated Cl^−^ secretion in CF patients. Purinergic agonists in addition to ATP such as UTP, UDP, and ADP also had the power to stimulate Cl^−^ secretion in CF and non-CF airway epithelial models [75]. In addition, adenosine receptors can also stimulate Cl^−^ secretion in airway epithelial cells by activating the cAMP/PKA signal transduction pathway and eventually CFTR [76, 77]. ATP signalling through purinergic P2Y receptors is effective in airway epithelia in inhibiting ENaC activity and initiating Ca^2+^-activated Cl^−^ secretion [78]. All functionally defined P2Y receptors are able to couple through the IP_3_ pathway consisting of activation of PLC increase in inositol phosphates and mobilization of Ca^2+^ from intracellular stores. In addition and secondary to the activation of the PLC, multiple signal transduction pathways including PKC, phospholipase A_2_, Ca^2+^ sensitive ion channels, and formation of endothelium derived relaxing factors have been shown to be involved in the responses to activation of native P2Y-receptors. Another function of the P2Y receptors is the activation of ciliary beat frequency. In hydrated airways, the rate of mucociliary clearance is determined by ciliary beat frequency and nonsaturating concentrations of ATP generates alternating Ca^2+^ signals in ciliated cells which in turn increases ciliary beat frequency [79, 80].

Pharmacological data has shown that the P2Y11 receptor is preferentially activated by ATP and is uniquely coupled to both the phosphoinositide and the cAMP pathways [81]. Evidence is available that ATP and ADP, two physiologic nucleotides that can be released into the extracellular space, are able to raise cAMP levels in native cells via activation of P2Y11 receptors. Those results provide a mechanism in addition to activation of P2Y2 or adenosine receptors, by which exogenous or endogenously related nucleotides can increase cellular levels of this important cyclic nucleotide. Given the evidence that a number of types of cells both release ATP and possess P2Y11 receptor, then nucleotide mediated activation of P2Y11 receptors provides a means for autocrine regulation of epithelial and other cell types. Activation of the P2Y11 receptor in different cell types has a number of different outcomes. For example, the P2Y11 receptor mediates the inhibition of neutrophil apoptosis, impaired endothelial cell proliferation or regulation of secretory function of pancreatic ductal cells by ATP [82–84].

### 4.2. Nucleotides Release by Pannexin Channel

The complex cellular composition of the airways that is ciliated cells and mucin-secretory goblet cells suggests that several mechanisms and pathways are involved in the release of nucleotides into the airways. Two general mechanisms for the release of ATP from cells have been proposed as vesicular release and channel-mediated release. While vesicular release of ATP is well documented, ATP release can also occur in the absence of vesicules. For example, human erythrocyte which is devoid of cytoplasmic vesicle can release ATP in low oxygen content or in response to shear stress [85]. Pannexins belong to the family of connexin channels that have been proposed as diffusion pathways for ATP release under various experimental conditions. The Pannexins primarily form oligomeric structures embedded in a single plasma membrane that when open provide a conduction pathway between cytosol and extracellular space. They are mechanosensitive and are highly permeable to ATP [86]. Exposure of the alveolar A549 cells to thrombin resulted in a strong ATP release response that was inhibited by the nonselective blockers of pannexin channels suggesting that ATP release from thrombin-stimulated lung epithelial cells occurs through pannexin channels [87]. A study by Ransford et al. 2009 showed ATP release induced by hypotonic shock of human bronchial epithelial cells was inhibited after silencing pannexin-1 (Panx1) via shRNA [88]. The large pores of Panx1 (the most studied pannexin channel) are permeable to ions, second messengers, and neurotransmitters such as ATP, IP_3_, and amino acids. Panx1 is also implicated in secretion of arachidonic acid and its metabolites and it is now widely regarded that Panx1 membrane channels are also involved in the extracellular mode of wave propagation. Panx1 channels open in response to mechanical stress or other stimuli such as depolarization and release ATP to the extracellular medium. ATP binding to purinergic receptors triggers an increase of cytoplasmic Ca^2+^ via the IP_3_ pathway. The Ca^2+^ increase is not restricted to the same cell but also includes cells within diffusion distance for the released ATP also stimulating cells that are coupled to the stimulated cell by gap junction channels permitting the flux of IP_3_.

The increase in Ca^2+^ can activate Panx1 channels and subsequent release of ATP provides a new source for extracellular ATP to reach more distant cells [86]. The application of micromolar concentrations of Ca^2+^ to the cytoplasmic side of Panx1 channels in excised membrane patches activated the channels at negative membrane potentials where the channels are normally closed [86].

### 4.3. Lipoxin A_4_ Increases the Airway Surface Liquid Height via P2Y11 Activation

**Figure 3:**
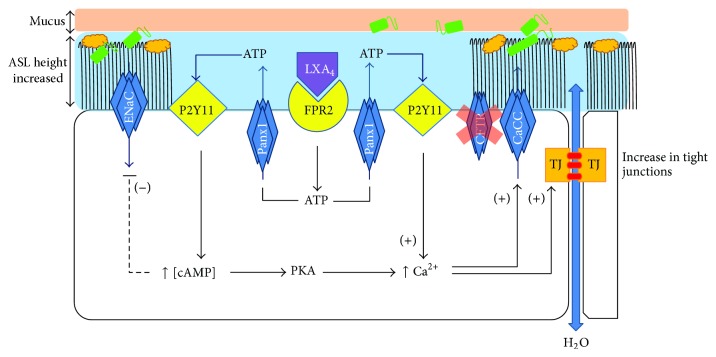
Lipoxin A_4_ enhances epithelial barrier integrity by stimulating an increase in airway surface liquid (ASL) layer height, epithelial repair, and tight junction formation. Stimulation of the FPR2 receptor by LXA_4_ induces an apical ATP release through the pannexin (Panx1) channel activating a purinoreceptor pathway. Activation of P2Y11 receptors stimulates chloride secretion out of the cell by calcium activated chloride channels (CaCC) and inhibition of sodium absorption by amiloride sensitive epithelial sodium channels (ENaC) which result in a restored ASL height in CF bronchial epithelial cells. The calcium signal induced by P2Y11 activation also stimulates epithelial repair and tight junction formation. Taken together, the physiological effects induced by LXA_4_ have the potential to delay the invasion of bronchial epithelial cells by bacteria (green and orange structures).

The mechanism by which LXA_4_ stimulates Ca^2+^-activated Cl^−^ secretion and ASL height increase has been elucidated. Higgins et al. reported that LXA_4_ induces an apical ATP release from non-CF and CF airway epithelial cell lines and CF primary cultures. This ATP release induced by LXA_4_ is completely inhibited by antagonists of the FPR2 receptor and Panx1 channels suggesting a major role of Panx1 in this effect. Furthermore, LXA_4_ induces an increase in intracellular cAMP and calcium, which are abolished by the selective inhibition of the P2RY11 purinoreceptor. Panx1 and ATP hydrolysis inhibition and P2RY11 purinoreceptor knockdown all abolish the increase of ASL height induced by LXA_4_. Inhibition of the A_2_b adenosine receptor does not affect the ASL height increase induced by LXA_4_, whereas the PKA inhibitor partially inhibits this response. Taken together this report provides evidence for a novel role of LXA_4_ in stimulating apical ATP secretion via Panx1 channel and subsequent P2RY11 purinoreceptor activation in airway epithelial cells leading to an ASL height increase (Figure 3).

## 5. Epithelial Repair in CF Airway

### 5.1. Altered Epithelial Repair in CF

In CF, recurrent infections and inflammatory insults result in damage to the airways and trigger the repair process [89]. Epithelial repair initially involves cell migration and cell proliferation to repopulate the injured area [90–92]. This process is then followed by differentiation of the epithelium [93]. Recent research suggests that epithelial repair as well as differentiation of the CF airway epithelium is downregulated or delayed [94–98]. More specifically, cell migration and proliferation both appear to be reduced during repair of CF bronchial epithelial cells compared to non-CF cells [99]. This delay in repair of the CF epithelium renders the lung more susceptible to ongoing bacterial infection and thus may trigger more epithelial damage [100].

### 5.2. Lipoxin A_4_ Regulates Airway Epithelial Integrity in CF Airway Epithelium

The lipid mediator LXA_4_ triggers epithelial cell migration and proliferation and thus plays a role in repair of epithelia including bronchial epithelium from patients with CF [22, 99, 101–104]. The effects of LXA_4_ in stimulating cell proliferation, cell migration, and wound repair are mediated by the apical ATP release and P2Y11 activation [105]. Stimulation of P2Y11 purinoreceptors induces calcium release and ERK phosphorylation, both of which play a key role in initiating cell proliferation and migration [106–113]. Furthermore, consistent with the role of potassium channels in two key processes of repair, migration, and proliferation in numerous cell types, the responses to LXA_4_ on the repair process are mediated by the downstream activation of K_ATP_ potassium channels [96, 97, 99, 114–117]. Additionally, LXA_4_ enhances airway epithelial tight junction formation which is a main factor of epithelial barrier integrity. LXA_4_ stimulates ZO-1, claudin-1, and occludin expression and trafficking at the apical membrane resulting in enhanced transepithelial electrical resistance in human airway epithelia [118] (Figure 2). Taken together, these effects of LXA_4_ on airway epithelial structure suggest the abnormal levels of LXA_4_ in CF airways may account for the reduced capacity for epithelial repair in CF.

## 6. Treatments of CF Airway Disease

### 6.1. Current Treatments and Opportunities

There is currently no treatment available that fully corrects the biochemical abnormality in CF and leads to a cessation of the typical pathobiology seen in the condition. Therapies to date have been centred on slowing the decline in pulmonary function over time to prolong survival. Medication is predominantly used to optimise nutrition (pancreatic enzymes, fat soluble vitamin supplementation), treat infection (oral, inhaled, and intravenous antibiotics), and facilitate effective mucociliary clearance (DNAse, hypertonic saline). Several anti-inflammatory approaches have been examined in CF; however, the ideal anti-inflammatory drug is not yet available [119]. A recent systematic review of the risks and benefits of inhaled corticosteroids in CF, examining evidence from 13 trials, concluded that there is insufficient evidence to establish whether they are beneficial in CF while it is established that ICS use can have adverse effects [120]. A systematic review of the efficacy of nonsteroidal anti-inflammatory drugs in CF concluded that treatment with high-dose ibuprofen was associated with a significantly lower annual rate of decline in lung function (especially in children); however, the adoption of ibuprofen into therapy has not been universally accepted [121]. Correcting the imbalance in fatty acid metabolism described in CF by supplementation of Docosahexaenoic Acid may be helpful, and efforts are ongoing to evaluate the potential therapeutic benefit [122].

Two promising avenues of therapy have recently emerged: small molecule correctors and gene therapy. The flagship small molecule corrector has been Ivacaftor (VX-770). This compound facilitates gating of defective CFTR where the cause of CFTR dysfunction is a gating mutation—predominantly G551D. This has been remarkably clinically successful but can be taken by only approximately 5% of patients worldwide [123, 124]. The manufacturers of Ivacaftor, Vertex Pharmaceuticals, are currently developing correctors for the commonest mutation Phe508del. Phase 2 trials of this compound have been shown to lead to positive changes in CFTR function, but not to the same degree as VX-770 [125]. Gene therapy was considered an obvious target for disease modifying treatment after the discovery of the CFTR gene; however initial attempts at this approach were unsuccessful, prompting a comprehensive review of the process of selection of endpoints, vectors, and delivery modes. A consortium in the UK has developed a comprehensive approach in this regard and will report shortly on multidose trials of gene therapy in individuals with CF [126]. A treatment approach capable of effectively preventing lung damage and decline in pulmonary function is currently absent despite the obvious hope relating to new developments.

For now, we continue to search for new and effective therapies to slow or prevent the decline in pulmonary function in CF.

### 6.2. Therapeutic Potential for LXA_4_ in the Treatment of CF Airway Disease

A variety of airway clearance therapies have been developed for patients with CF [127, 128]. Thus identification of agents, particularly endogenous biologicals that stimulate non-CFTR Cl^−^ secretory pathways and promote ASL height recovery while providing anti-inflammatory effects are likely to be of therapeutic benefit in improving mucociliary clearance in patients with CF. The effect of LXA_4_ inhalation has been evaluated in a pilot study of eight asthmatic and healthy adult subjects. The challenge was tolerated, had no adverse effect on pulse or blood pressure, and demonstrated favourable effects on specific airway conductance [129].

In conclusion, the discovery of the multiple impacts of LXA_4_ in restoring bronchial epithelium ion transport, in enhancing ASL height, in restoring epithelial barrier function, and in reducing inflammation might provide significant advance in treatment of the CF airway disease (Figure 3).
